# Immunomodulatory role of decidual prolactin on the human fetal membranes and placenta

**DOI:** 10.3389/fimmu.2023.1212736

**Published:** 2023-06-09

**Authors:** Pilar Flores-Espinosa, Isabel Méndez, Claudine Irles, Andrea Olmos-Ortiz, Cecilia Helguera-Repetto, Ismael Mancilla-Herrera, Daniel Ortuño-Sahagún, Vincent Goffin, Verónica Zaga-Clavellina

**Affiliations:** ^1^ Departamento de Inmunobioquímica, Instituto Nacional de Perinatología Isidro Espinosa de los Reyes, Ciudad de México, Mexico; ^2^ Departamento de Neurobiología Celular y Molecular, Instituto de Neurobiología, Universidad Nacional Autónoma de México (UNAM), Campus UNAM-Juriquilla, Querétaro, Mexico; ^3^ Institut National de la Santé et de la Recherche Médicale (INSERM) U978, Université Sorbonne Paris Nord, Unité de Formation et de Recherche (UFR) Santé Médecine et Biologie Humaine (SMBH), Bobigny, France; ^4^ Departamento de Infectología e Inmunología, Instituto Nacional de Perinatología Isidro Espinosa de los Reyes, Ciudad de México, Mexico; ^5^ Laboratorio de Neuroinmunobiología Molecular, Instituto de Investigación en Ciencias Biomédicas, Universidad de Guadalajara, Guadalajara, Mexico; ^6^ Université Paris Cité, Institut National de la Santé et de la Recherche Médicale (INSERM), Unité Mixte de Recherche (UMR)-S1151, CNRS Unité Mixte de Recherche (UMR)-S8253, Institut Necker Enfants Malades, Paris, France

**Keywords:** immune privilege, decidual prolactin, pregnancy, maternal-fetal interface, fetal membranes, placenta, preterm labor, innate immunity

## Abstract

The close interaction between fetal and maternal cells during pregnancy requires multiple immune-endocrine mechanisms to provide the fetus with a tolerogenic environment and protection against any infectious challenge. The fetal membranes and placenta create a hyperprolactinemic milieu in which prolactin (PRL) synthesized by the maternal decidua is transported through the amnion-chorion and accumulated into the amniotic cavity, where the fetus is bedded in high concentrations during pregnancy. PRL is a pleiotropic immune-neuroendocrine hormone with multiple immunomodulatory functions mainly related to reproduction. However, the biological role of PRL at the maternal-fetal interface has yet to be fully elucidated. In this review, we have summarized the current information on the multiple effects of PRL, focusing on its immunological effects and biological significance for the immune privilege of the maternal-fetal interface.

## 1 Introduction

Human pregnancy represents a unique immune-endocrine state that allows a semi-allogeneic fetus to grow and develop in a tolerogenic environment created and protected by the maternal-fetal interface. The interface between the transformed endometrium (decidua) and the blastocyst is the scenario in which diverse critical immune-endocrine crosstalk between maternal and fetal cells develop to establish immune privilege during pregnancy. Tolerance is required to avoid fetal rejection, and at the same time, the maternal immune system should be ready to respond efficiently to any infection or pathological inflammation (1, 2).

As pregnancy progresses, the maternal-fetal interface increases and the placenta and fetal membranes expand their close contact with the decidua. Therefore, the immunological balance between fetal and maternal cellular responses is critical for a successful outcome (3).

**Figure 1:**
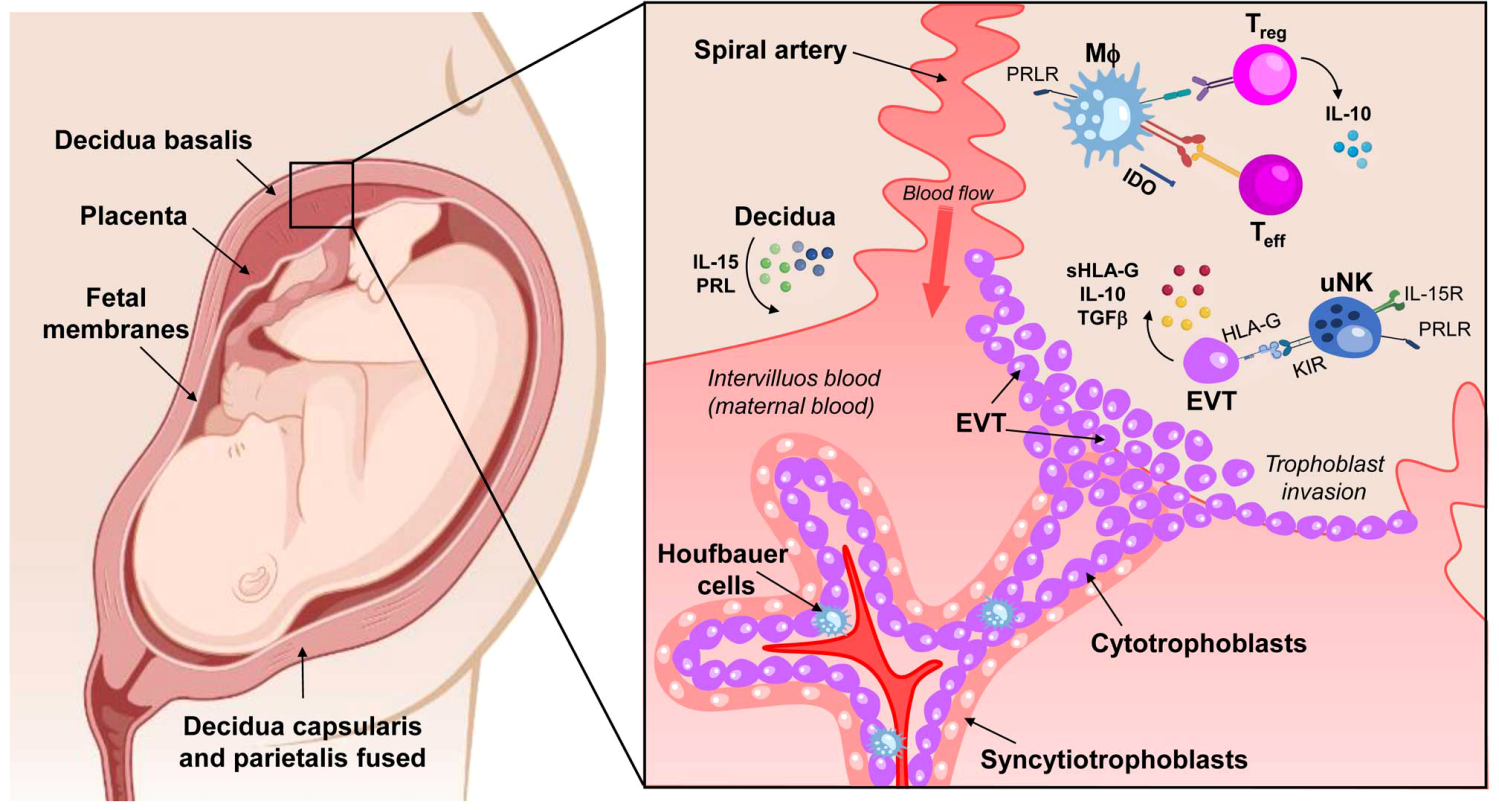
Schematic of a maternal-fetal unit composed of decidua, placenta, and fetal membranes. There are three types of decidua, depending on their location and cellular interactions: decidua basalis in contact with the placental bed, decidua capsularis in direct contact with the chorionic layer of the fetal membranes, and decidua parietalis, lining the uterine cavity and eventually fusing with the decidua capsularis. The close contact between these tissues involves the interaction between maternal immune cells and fetal cells (trophoblasts). The decidua cells produce IL-15 and PRL to stimulate uterine NK cells (uNK) proliferation. The uNk cells recognize HLA-G expressed by the extravillous trophoblast (EVT), triggering a tolerogenic response. The EVT is the active agent in trophoblast invasion, but it also expresses IL-10 and TGF-β, anti-inflammatory factors that reinforce the tolerogenic environment in the decidua. In macrophages (MΦ), PRL mediates IDO expression by promoting the synthesis of kynurenine, which is cytotoxic to T effector lymphocytes (Teff) and NK cells. In addition, PRL also appears to be involved in the differentiation of T regulatory cells (Treg) cells, an essential source of IL-10. IL-15R, receptor of IL-15; PRL, Prolactin; PRLR, Prolactin receptor; TGF-β, Transforming Growth Factor β; KIR, Killer-cell immunoglobulin-like receptors; IDO, Indoleamine 2,3-dioxygenase. Created in BioRender.com.

The process of differentiation between the chorionic sheet of fetal membranes, which is composed of connective tissue and in which the fetal blood vessels and extravillous trophoblasts cells are located, is accompanied by changes in the surrounding maternal tissues. Depending on its spatial relationship to the implanting chorionic sac, the decidua is divided into several segments. The decidua below and lateral to the blastocyst or later the one below the placenta is the basal decidual (decidua basalis). Once implantation is complete, the decidua closes over the blastocyst, this protruding layer is the capsular decidua (decidua capsularis). All parts of the decidua that line the uterine cavity without being in contact with the blastocyst are called the parietal decidua (decidua parietalis) (4). (**Figure 1**).

Several studies conducted between 1970 and 1980 provided the first evidence for the role of the hormone prolactin (PRL) in the maternal-fetal unit, which is essential for establishing and maintaining a pregnancy (5–7). These works also showed that pregnancy is a state of physiological hyperprolactinemia.

PRL is classified as a pleiotropic immune-neuroendocrine hormone and an autocrine/paracrine factor that regulates more than 300 biological functions, including immune regulation, metabolism, angiogenesis, and osmoregulation (8). In addition, PRL plays an essential role in reproductive processes, such as sex steroid production, blastocyst implantation, placentation, and lactation (8–12). In rodents and other mammals, PRL regulates maternal behavior (13) and maintenance of the corpus luteum (14, 15).

Pioneering studies have shown that PRL-knockout (KO) female mice have irregular estrous cycles and do not become pregnant when mated with fertile males. Although the egg can be fertilized, implantation does not occur (16). Studies using PRL receptor (PRLR)-KO mice have shown that females also fail to become pregnant because they have multiple reproductive defects before implantation, which explains the poor survival of embryos (17). In addition, PRL-KO and PRLR-KO mice are entirely infertile because the corpus luteum relies on PRL secretion to induce progesterone (P4) synthesis and endometrial vascularization to support implantation (17).

Despite the relatively well-known effects of PRL on blastocyst implantation and fertilization, there is no recent research on the recently described immunomodulatory functions of PRL, focusing on its effects on human fetal membranes and the placenta. In this review, we present the findings of our group and others aiming at understanding the regulatory signals modulated by PRL to promote an immunotolerant state in the essential tissues that protect, nourish, and shelter the fetus.

## 2 Regulation of extra pituitary PRL synthesis

In addition to the pituitary gland, tissues such as the uterus, especially decidualized endometrial stromal cells and myometrial cells, the ovaries, and the immune system, also produce extra pituitary PRL that exerts mainly local autocrine and paracrine functions (18).

Remarkably, the classical regulators of PRL synthesis, i.e. dopamine, thyrotropin-releasing hormone (TRH), and pituitary PRL *per se*, have shown no effect on extra pituitary PRL production *in vitro* (18). Extra pituitary synthesis and secretion mainly depend on the source cell type and their associated microenvironment. For example, P4 is the primary regulator of PRL release in the decidua and mammary glands but does not affect PRL secretion in adipose tissue (19). In contrast, calcitriol influences PRL production in the decidua and lymphocytes (20, 21). Furthermore, decidual PRL expression is inhibited by different inflammatory cytokines, such as interleukin (IL)-2 and tumor necrosis factor-alpha (TNF-α), as well as factors with anti-inflammatory properties such as transforming growth factor beta (TGF-β) (22–24).

**Figure 2:**
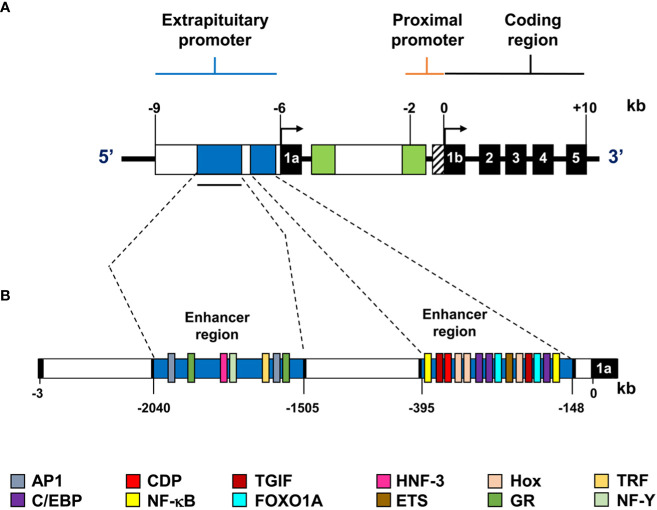
Representation of the extra pituitary promoter of the human PRL gene. **(A)** The extra pituitary and proximal promoters and the coding region are shown; exons are represented by black boxes with corresponding exon numbers (1a, 1b, 2-5), and transcription start sites are indicated by arrows. **(B)** Diagram of the alternative human PRL promoter. This superdistal promoter is over 2.0 kb long and contains two enhancer regions shown in blue. The consensus sequences for the transcription factors are shown in different colors.

Transcriptional regulation of PRL is controlled by two promoters, a proximal one associated with pituitary expression and a distal one associated with extra pituitary expression (**Figure 2A**). The distal promoter is more than 2 kb-long and includes two enhancer regions that contain binding sites for CCAAT/enhancer-binding protein beta (C/EBPβ), Forkhead Box O1A (FOXO1A), Nuclear factor kappa-light-chain-enhancer of activated B cells (NF-kB), Activator protein 1 (AP-1) responsive elements, among others (18, 25) (**Figure 2B**). Notably, activation of the cAMP/PKA pathway induces extra pituitary PRL promoter activity in human decidual cells through the transcription factors CREB and C/EBP (25, 26). The transcription factors HoxA-11 and FOXO1A also physically and functionally interact to upregulate PRL gene expression in the decidualized endometrium (27, 28). Also, Ets-1 transcription factor is critical for basal expression of decidual PRL. In contrast, PRL expression is not affected by this factor in non-decidualized endometrial stromal or fibroblast cells (29, 30), indicating tissue-dependent control mechanisms.

## 3 Dynamics of PRL production during human pregnancy and its compartmentalized effects on the decidua, placenta, and fetal membranes

**Figure 3:**
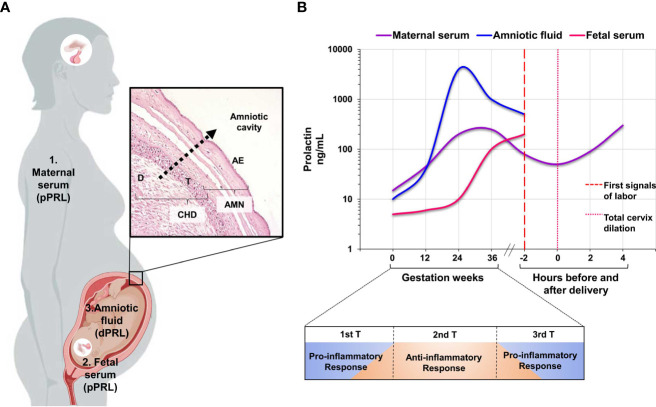
Compartmental release of PRL during human pregnancy. **(A)** The figure shows the three compartments: maternal, fetal, and decidual compartments, which are independently regulated during pregnancy, and the transport of PRL from the decidua to the amniotic cavity through fetal membranes. **(B)** The graph shows the patterns of PRL secretion in the three independent compartments. Maternal serum and amniotic fluid show the increase in PRL concentration during the second trimester of pregnancy and the subsequent decrease in concentration at the onset of labor. Finally, the relationship between the pattern of PRL secretion and the three stages of pregnancy, as well as the immune response at each of these stages, supports the role of PRL as a regulator of the immune environment during a successful gestation. AMN, amnion-fetal side; CHD, choriodecidua-maternal side; D, decidual cells; T, trophoblast cells; AE, amniotic epithelium. The graph is taken from references 6, 47-49. Created in BioRender.com.

During pregnancy, the major sources of PRL are the maternal pituitary gland, decidua, and fetal pituitary gland, in which PRL is independently regulated. PRL detected in maternal serum originates from her pituitary gland; on the other hand, the increase of PRL begins in the 10th week of gestation (31). However, PRL accumulated in the amniotic fluid (AF) is mainly attributed to the decidual cells (32–34). After synthesis and secretion, decidual PRL is transported through fetal membranes into the amniotic cavity, where it accumulates significantly (7, 35, 36) (**Figure 3A**).

The observed changes in the PRL release profile in the three independent compartments suggest an immunomodulatory role of this hormone at different stages of pregnancy (**Figure 3B**), which will be discussed in the following sections with emphasis on the fetoplacental unit. The specific secretion of PRL by the decidua and the changes in its concentration in the amniotic cavity and maternal serum support its role as an essential modulator of tolerance and are relevant to the onset of labor.

### 3.1 Circulating PRL level in maternal serum

In nonpregnant women, serum PRL levels are below 23 ng/mL, whereas a hyperprolactinemic state characterizes pregnancy. PRL increases 3- to 8-fold in the first trimester and is continuously released until it reaches 250 ng/mL in the third trimester (7, 37). Before the onset of labor, maternal serum PRL levels gradually decrease to about 60 ng/mL; this low concentration has been clinically associated with the first signs of labor and complete cervical dilatation (6). After delivery, PRL levels remain elevated, allowing lactotrophic hyperplasia and breast development for breastfeeding (38).

### 3.2 Decidual PRL and its autocrine functions

The human decidua synthesizes and secretes a PRL immunologically, chemically, and biologically indistinguishable from the pituitary gland (32, 39).

In the past four decades, P4 stimulus for decidualization of endometrial stromal cells during the luteal phase of the menstrual cycle has been shown to be an essential step to initiate PRL production by the decidua (40, 41). Kinoshita et al. reported a 2-fold higher content of PRL in the decidua before labor (370.3 ± 67.7 ng/mg wet weight) than at labor (196.8 ± 47.2 ng/mg wet weight) (42).

During pregnancy, the expression of PRLR isoforms in various female reproductive tissues, including the ovary, corpus luteum, and decidua, is hormonally and temporally regulated (43–45). The major PRLR isoforms in the decidua are short and long (PRLR-L and PRLR-S). They have also been observed in the amniotic epithelium, chorionic cytotrophoblasts, and syncytiotrophoblasts (45–48). Jak-2 is autophosphorylated after PRLR-L activation and generates coupling sites for other substrates containing Src homology domain 2 (SH2), such as the STAT proteins (STAT5a and 5b), through tyrosine phosphorylation, dimerization, and subsequent translocation to the nucleus to regulate transcription of various target genes [for a detailed review of PRL signaling pathways, see Kavarthapu and Dufau (49)]. Although PRLR-S cannot activate Jak2/Stat signaling pathways, it can trigger MAPK-dependent PI3K signaling pathways that contribute to normal follicular development via activation of the phosphatase DUPD1 in the ovary and decidua (44) Because MAPK is a key factor in follicular development and normal decidua formation, PRLR-S appears to play an essential role in these stages of reproduction.

Decidual PRL has an autocrine effect and regulates the decidua’s hormonal, immunological, and surveillance functions. For example, PRL significantly affects decidua regression, a fundamental event that occurs through apoptosis at the end of gestation (50). Animal models have helped clarify that PRL has antiapoptotic effects via PRLR-L, which downregulates caspase-3 mRNA levels independently of Jak2 activation (51). Thus, the loss of PRLR-L that occurs in the mesometrial and antimesometrial decidua correlates temporally with the cell death that occurs in this tissue during late gestation (51). Consistent with this, PRLR-KO mice exhibit high levels of apoptosis, further supporting the potent antiapoptotic effect of PRL (52).

As the diameter of the chorionic sac increases, the capsular decidua degenerates focally so that the outer surface of the smooth chorion is adjacent to the uterine cavity at these sites. Between weeks 15 and 20 after conception, the chorion, along with the remainder of the capsular decidua, fuses locally with the parietal decidua, thereby largely obliterating the uterine cavity. From this point on, the chorionic leaflet is in contact with the decidua of the uterine wall over almost its entire area and can function as a paraplacental exchange organ (4).

The decidua basalis contributes to the formation of the placental base plate, and this rigid part of the decidua directs the interstitial invasion of trophoblasts to form the anchoring columnar villi. In contrast, the capsular decidua is laxer and, because of its location, represents the major source of PRL in the amniotic fluid before it fuses with the parietal decidua (53).

There is no evidence of differential PRL secretion between the three decidua types, but increased *in vitro* PRL production and secretion by 2nd trimester decidua explants (1450 ng/g of tissue) compared with 1st and 3rd trimester explants (992 and 728 ng/g of tissue respectively) has been described (54).

Similar to the pituitary gland, the decidua can secrete glycosylated PRL, such that 30-50% of PRL in amniotic fluid is glycosylated (55, 56). Glycosylation of PRL enhances or decreases its activity in a tissue-specific manner (57–59). The effects of glycosylation directly on placental and fetal membranes remain to be elucidated.

The cDNA sequence of decidual PRL has four silent changes in its nucleotides, two of these changes are in the third position of the amino acid codons and the other two are in the 3’-untranslated region, resulting in an amino acid sequence identified in decidual and pituitary PRL (60, 61). Decidual PRL has also been found to have the same biological and immunological activity as pituitary prolactin (61, 62).

Moreover, this hormone inhibits the production of decidual IL-6, a critical pro-inflammatory signal at the feto-maternal interface, and the production of 20α-hydroxysteroid dehydrogenase (20α-HSD), preventing P4 catabolism (63); the control of the inflammatory environment by PRL contributes to the maintenance of fetal development in an immunotolerant scenario (64, 65).

Finally, decidual PRL is intimately involved in the control of angiogenesis. On the one hand, PRL can directly stimulate endothelial cell proliferation and migration in an autocrine and paracrine manner (66). Moreover, in an *in vivo* model of chick embryo chorioallantoic membrane, PRL stimulated vascular density and column formation characteristic of intussusceptive angiogenesis in embryogenesis (67). This allows speculation about a direct role of PRL in the regulation of placental angiogenesis. However, PRL may stimulate angiogenesis indirectly by promoting the synthesis of other angiogenic factors such as fibroblast growth factor (bFGF) and vascular endothelial growth factor (VEGF) in the decidua and immune cells (68–70).

On the other hand, PRL can also acquire antiangiogenic properties after proteolysis by generating PRL fragments (5.6-18 kDa) called vasoinhibins (15, 71). During pregnancy, decidual PRL can be cleaved by captesin-D produced by the placenta (72). Vasoinhibins do not compete for binding to PRL-R. *In vitro* studies show that these peptides bind to the fibrinolytic inhibitor plasminogen activator inhibitor-1 (PAI-1), the urokinase-type plasminogen activator PAI-1 (uPA) and the uPA receptor (uPAR) to form a ternary complex on the surface of endothelial cells. By forming this complex, vasoinhibins regulate ERK1/2 and NFkB signaling to exert their profibrinolytic and antiangiogenic functions (73).

### 3.3 Biological functions of PRL in the human placenta

The placenta is a temporary organ that grows and develops during pregnancy and disappears after delivery. This extraembryonic tissue is in constant cellular communication with the maternal decidua and blood, as well as with the fetal membranes, AF, and fetus. This bidirectional feto-maternal communication is critical for the regulation of several essential placental functions for growth and monitoring of the developing fetus, as well as for the placental establishment and protection of the fetus from microbial challenges (74). PRL levels detected in the intervillous space are similar to maternal plasma levels (131.6 ± 64.3 ng/mL); in the umbilical cord, PRL levels are higher than in the placenta (243.8 ± 86.1 ng/mL) (75).

Considering that the placenta is an essential site for the synthesis and action of this hormone (mainly in villous and extravillous cytotrophoblasts (76, 77) two main axes of placental development are now studied: trophoblast migration and invasion and control of fetal inflammatory responses.

**Figure 4:**
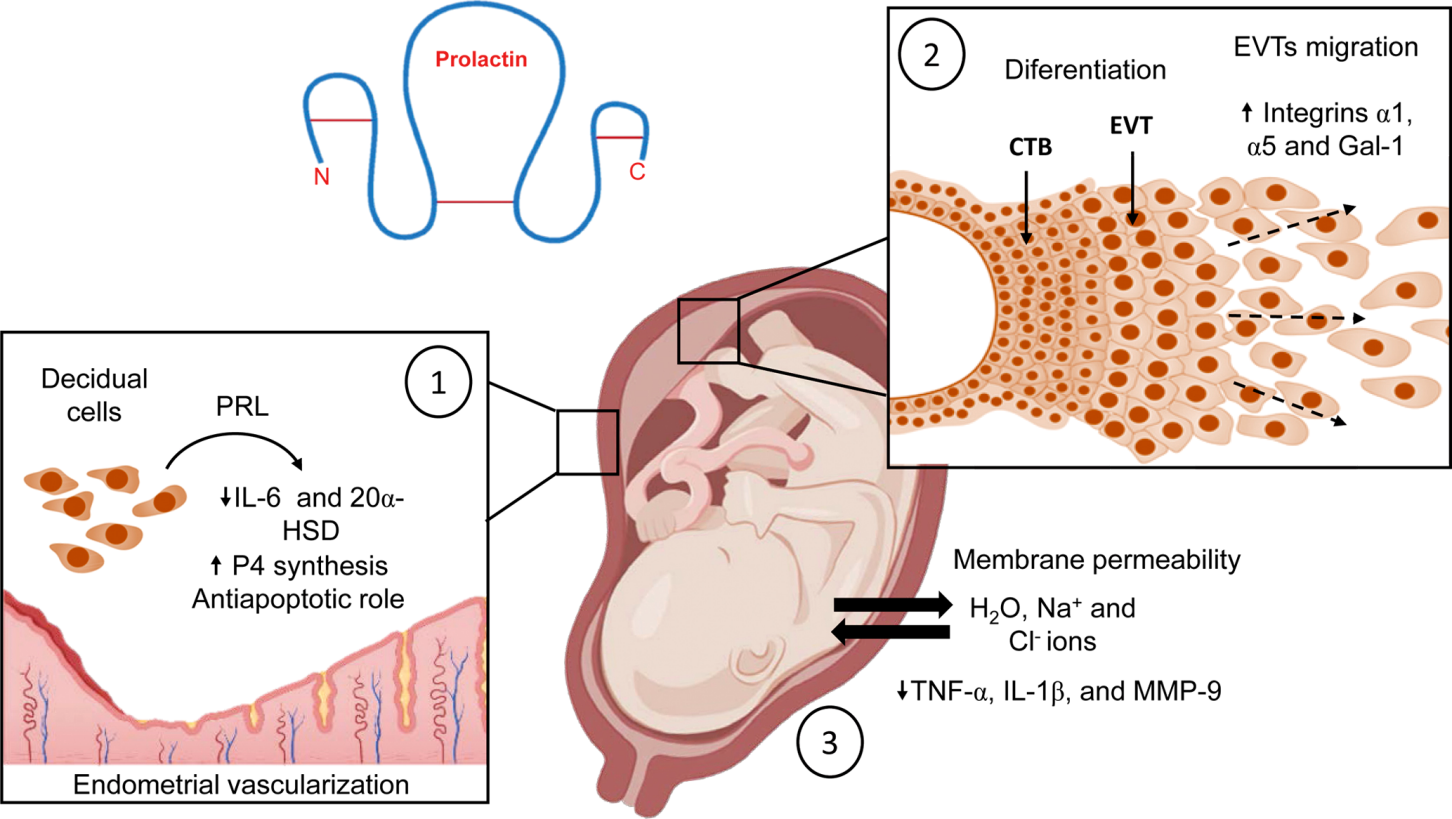
Biological effects of PRL at the maternal-fetal interface during normal pregnancy. (1) PRL inhibits decidual production of IL-6, maintains P4 production, regulates endometrial vascularization, and stimulates proliferation of endothelial cells to support implantation; additionally, it has antiapoptotic properties. (2) In the placenta, this hormone stimulates the differentiation of EVTs into trophoblasts with an invasive profile, also PRL leads migration of columnar EVTs into the maternal spiral arteries. (3) PRL modulates fetal membrane permeability to maintain the water-electrolyte balance and adequate amniotic fluid volume in the amniotic cavity. In addition, PRL downregulates the expression of TNF-α, IL-1β, and MMP-9 to maintain the immune privilege of the amniotic cavity. Created in BioRender.com.

In the first trimester of gestation, various decidual factors stimulate the differentiation of extravillous trophoblasts (EVT) into invasive trophoblasts, leading to the migration of columnar EVTs into the maternal spiral arteries and decidua (78). Among the factors produced by the decidua, PRL triggered an invasive trophoblast phenotype *in vitro*, highlighting its involvement in placental angiogenesis, trophoblast growth, and migration (77). Even in the early stages of the blastocyst, PRL promotes trophoblast cell migration *in vitro* (79). Immunohistochemical analysis revealed that columnar cytotrophoblasts abundantly express PRLR and respond to its ligand with increased expression of integrins α1 and α5, and galectin-1, all markers of endovascular and interstitial EVTs (77). Therefore, PRL is an additional factor to consider in controlling the epithelial-mesenchymal transition of EVTs, and in regulating early trophoblast invasion into the decidua (**Figure 4**).

**Figure 5:**
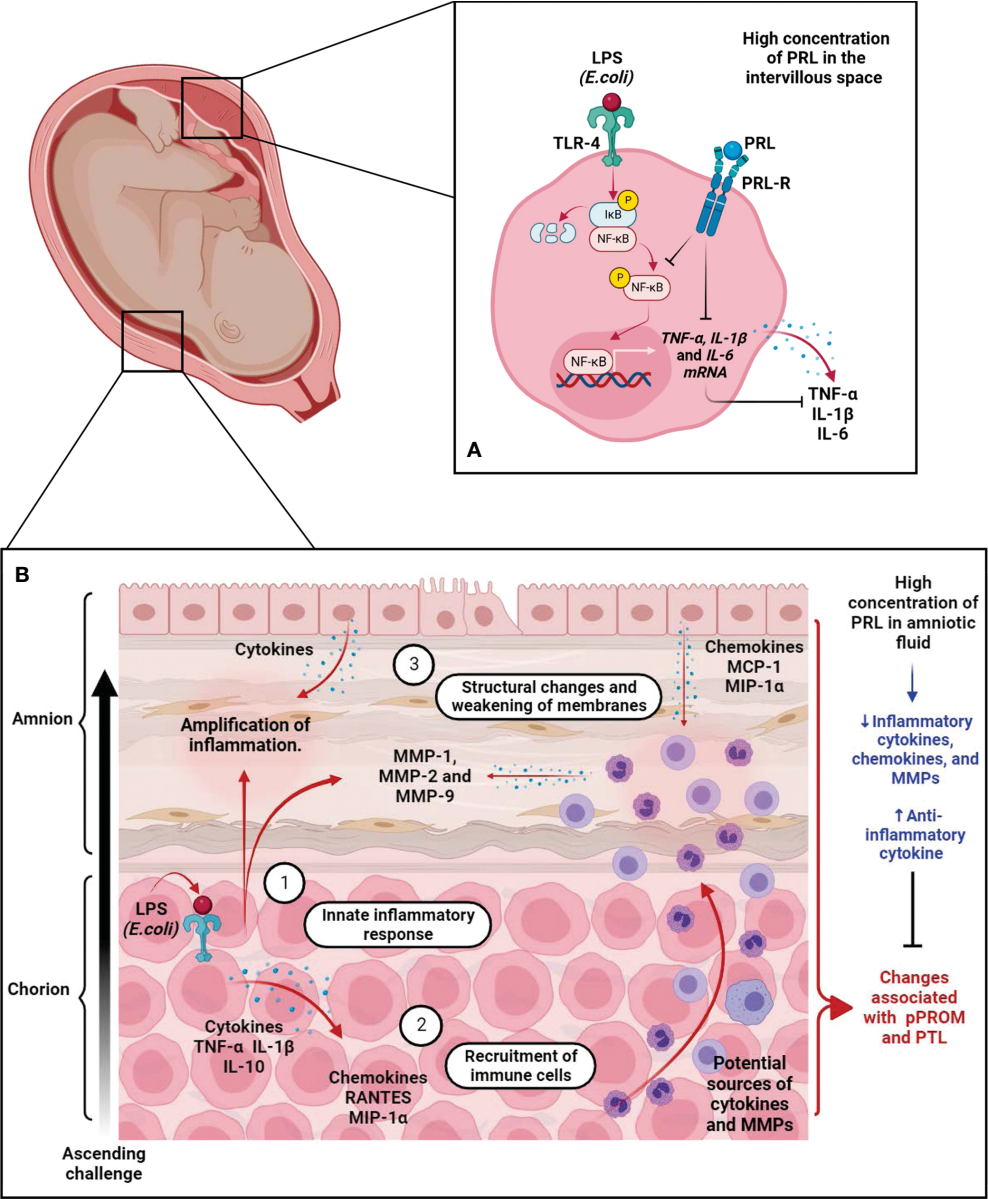
The immunomodulatory effects of PRL on the placenta and fetal membranes during immune challenge. **(A)** In the placenta, we show that the immunomodulatory effects of PRL are mediated in part by downregulation of TLR-4 expression in the trophoblast and subsequent phosphorylation of NFkB, thereby reducing the synthesis and secretion of pro-inflammatory factors. **(B)** Fetal membrane response during the ascending immune challenge. (1) Recognition of PAPMs such as LPS by TLR-4 expressed by trophoblasts in the choriodecidual region triggers an inflammatory response leading to secretion of pro-inflammatory cytokines and chemokines, which trigger infiltration of leukocytes into the site of inflammation/infection. (2) These chemoattractant cells release pro-inflammatory factors, including cytokines, chemokines, and ROS, which exacerbate the inflammatory process. (3) Additionally, these cells release MMPs, whose collagen activity causes structural changes in the connective tissue of fetal membranes, leading to their weakening and eventual rupture. These processes are part of the mechanisms leading to PTL and pPROM. Interestingly, experimental data from our group demonstrated that PRL attenuates the innate immune response of fetal membranes during this pathological scenario. These results support the hypothesis that PRL compartmentalization at the maternal-fetal interface is a critical factor in maintaining immune privilege during intrauterine infection, one of the major risk factors compromising pregnancy continuity. Created in BioRender.com.

Regarding the immunomodulatory properties of PRL, our research group has demonstrated that PRL can attenuate several markers of the inflammatory response in human fetal membranes stimulated with lipopolysaccharides (LPS) from E. coli (48, 80–82). In addition, we have investigated the immunological properties of PRL in the human placenta, particularly in villous trees at birth. As in human fetal membranes, PRL reduced the inflammatory response of placental villi explants stimulated with LPS. Moreover, we found that the anti-inflammatory mechanism of PRL in the placenta is mediated by a minor activation of the Toll-like receptor 4 (TLR4)/NFκB pathway and, consequently, a decreased secretion of TNF- α, IL-1β, and IL-6 by the villi (83). In addition, mononuclear cells isolated from the umbilical cord, placenta, and maternal blood respond to PRL with a reduction in IL-1β secretion under inflammatory conditions (84). Interestingly, this attenuation of the inflammatory milieu by PRL is observed mainly in feto-maternal tissues. In contrast, in other peripheral cells, such as macrophages and T lymphocytes, this hormone promotes the synthesis of a large battery of pro-inflammatory cytokines (85–87). Therefore, tissue-specific downregulation of pro-inflammatory cytokines by PRL in the maternal-placental-fetal unit may benefit fetal membrane integrity and maintenance of immune privilege during pregnancy (**Figure 5A**).

### 3.4 PRL accumulation in AF and its effects on human fetal membranes

PRL reaches a peak of 3,000 – 4,000 ng/mL in the AF at about 22 weeks of gestation; this concentration is the highest reached in life. After that, the PRL concentration begins to decline, reaching 350-500 ng/mL at 37 weeks gestation, when the pregnancy is considered almost complete, and these low levels are associated with the first signs of labor (7).

Fetal membranes are a transient tissue composed of two adjacent tissues: the amnion, which is in contact with the fetus and AF, and the chorion, which fuses with the maternal decidua. They are connected by several layers of connective tissue that give them strength and resistance (88).

The fetal membranes form a selective barrier with mechanical and immunological properties that ensure fetal protection throughout pregnancy: 1) They regulate the influx of water, electrolytes, and molecules into the amniotic cavity to maintain a homeostatic environment; 2) They support the mechanical stress of fetal movements and the volume of AF, and have a cellular and collagen remodeling mechanism to maintain their structure and function; 3) They produce signals (anti-inflammatory cytokines, immunomodulators, and antimicrobial peptides) to maintain immunological tolerance; 4) They act as a physical barrier to prevent pathogens from entering the amniotic cavity; 5) These tissues monitor the environment to detect potential signs of danger (pathogens or harmful endogenous molecules), and 6) They may also secrete cytokines, chemokines, and antimicrobial peptides as a defense mechanism (89).

Fetal membranes are a target organ for PRL because they express PRLR (46, 48). The most extensive effect described for decidual PRL is osmoregulation in fetal membranes. This hormone controls the exchange of electrolytes and modulates the permeability of fetal membranes to maintain the proper volume of AF. PRL decreases water flux from the maternal to the fetal compartment to allow the increase of Na^+^ and Cl^-^ ions in AF (90, 91). Clinically, low expression of PRLR in fetal membranes is associated with excessive AF volume in the amniotic cavity. This obstetric problem is known as polyhydramnios.

Consequently, the osmotic imbalance leads to greater mechanical stress on the membranes and increases the risk of fetal-membrane rupture, which may alter the normal course of pregnancy (92). In addition, *in vitro* studies have shown that PRL regulates basal production of inflammatory modulators associated with fetal membranes rupture at birth. Exposure of human fetal membranes to PRL decreased the production of prostaglandin E2 in fetal membranes (91). Furthermore, our research group demonstrated that PRL decreased basal mRNA expression and secretion of TNF-α, IL-1β, and matrix metalloproteinase (MMP) -9 by human fetal membranes in culture (80). This evidence suggests that high concentrations of PRL in AF are essential for maintaining an anti-inflammatory environment in the amniotic cavity where the fetus develops. These data support the hypothesis that decreasing PRL levels in the last weeks of gestation causes a switch from an anti-inflammatory environment to a pro-inflammatory milieu in the amniotic cavity. In this context, the onset of labor is considered a pro-inflammatory process. In addition, recent evidence suggests that inflammatory signals from fetal membranes act as alarm signals that spread to the uterus, decidua, and placenta and contribute significantly to the inflammatory cascade that initiates labor (93, 94).

#### 3.4.1 The role of PRL in preterm labor and preterm premature rupture of membranes (pPROM)

pPROM is a disease of fetal membranes that occurs as rupture of membranes before 37 weeks of gestation in the absence of another labor mechanism. This pathologic condition precedes 40-50% of cases of preterm labor (PTL) (95, 96), which in turn is the leading cause of neonatal morbidity and mortality (97).

Several clinical and experimental studies indicate that ascending lower genital tract infections and sterile inflammation in the amniotic cavity are risk factors for developing pPROM (98, 99). The amnion and choriodecidua express Toll Like Receptors (TLRs), innate immunity receptors that enable them to recognize Pathogen Associated Molecular Patterns (PAMPs) or Danger Associated Molecular Patterns (DAMPs), also known as alarmins. Activation of TLRs by PAMPs or DAMPs initiates the NF-kB signaling pathway, which upregulates the synthesis and secretion of pro-inflammatory cytokines and chemokines (100–105). This asynchronous inflammatory response disrupts the continuity of normal pregnancy (106, 107).

Our recent research demonstrated that PRL modulates the innate immune response deployed by fetal membranes during a pathological inflammatory process. Using an ex vivo model recreating the scenario of an ascending infection, we demonstrated that only high concentrations of PRL, corresponding to those in AF, selectively reduce LPS-induced TNF-α and IL-1β release in the choriodecidual region but have no effect on IL-6 and IL-10 (48).

This selective regulation is interesting because IL-10 is among the anti-inflammatory factors that promote the induction and maintenance of allograft tolerance at the maternal-fetal interface (108, 109). In contrast, TNF-α and IL-1β are pro-inflammatory cytokines that induce preterm labor (110). These cytokines also induce secretion and activity of MMP-2 and MMP-9 in fetal membranes, critical factors in collagenolytic processes leading to the weakening of fetal membrane and pPROM (111).

We also examined the effect of PRL on these collagenolytic enzymes. We found that PRL decreased the release of MMP-1, MMP-2, and MMP-9 and the collagenolytic activity of MMP-2 and MMP-9 in tissue extracts of fetal membranes induced by LPS (82). Collagen type IV is the main substrate of MMP-2 and MMP-9 and forms the scaffold for the interstitial collagen fibers (type I, II, and III), which are the substrates of MMP-1 (111). These collagen fibers are the major components of the basal lamina that supports the amniotic epithelium. The increased secretion and activity of these MMPs due to the inflammatory response leads to an irreversible change in the structure of the membranes and impairs their function as a physical and immunological barrier (112–114). At the histological level, PRL prevents the structural changes induced by LPS in the inner layers of the amnion and preserves the structural integrity of the tissue (82).

Finally, considering the infiltration of leukocytes into this tissue as part of the defense mechanisms, we investigated the effect of PRL on chemotactic factors released from fetal membranes. Our results showed that PRL decreased the release of Monocyte Chemoattractant Protein-1 (MCP-1), Macrophage Inflammatory Protein-1 Alpha (MIP1-α), and Regulated upon Activation, Normal T Cell Expressed and Presumably Secreted (RANTES), which are induced by LPS in fetal membranes (81). These chemokines selectively attract monocytes, macrophages, T cells, and NKs, potential sources of pro-inflammatory and degradative modulators that exacerbate the deleterious environment in the amniotic cavity (115, 116). Moreover, PRL reduced T-cell migration in chemotaxis assays in response to media conditioned by the amnion and choriodecidua (117).

In summary, PRL selectively regulates the first wave of inflammatory factors (cytokines and chemokines), decreases the second wave of degradative factors, favors an environment compatible with immune privilege, and partially controls the deleterious response during infection (**Figure 5B**). These results provide further evidence for the role of PRL as an immunomodulator in these extraembryonic tissues, whose functions are also essential for pregnancy health. It would be interesting to explore the mechanistic aspects of PRL that influence membrane functions in different contexts, such as senescence and oxidative stress, which are involved in the sterile inflammation of fetal membranes.

### 3.5 Serum levels of fetal PRL

In the fetal pituitary, PRL levels greater than 2 ng per total pituitary protein content are detectable around week 10 (31). Fetal PRL serum levels remain low (19 ng/mL) until 30 weeks gestation and then increase rapidly to reach 300 - 500 ng/mL by the end of pregnancy (18). Fetal PRL is associated with early chondrogenesis and central nervous system development; it is also involved in insulin production before and after birth (31, 118).

### 3.6 Conclusions and future perspectives

This review summarizes the regulation of decidual PRL production and its central role in the complex immune-endocrine responses at the maternal-fetal interface. PRL levels fluctuate throughout pregnancy in the different compartments of the feto-maternal interface and show the highest concentration around the second trimester of pregnancy, which coincides with the period of the anti-inflammatory response. Then, a decrease in PRL concentration coincides with the environmental shift toward a pro-inflammatory response, coinciding with the lowest PRL levels and the onset of labor.

PRL promotes maternal-fetal tolerance throughout pregnancy and regulates trophoblast growth and placental metabolism, amniotic cavity osmotic pressure, and fetal membrane innate immune responses. PRL is thus an essential component of the endocrine clock that controls the adaptations required at each stage of pregnancy and ultimately drives the activation of the decidua and fetal membranes to ensure successful delivery.

The immunomodulatory role of PRL in the mechanisms of fetal membrane senescence and its role in adverse events such as pPROM and PTL may be part of the next experimental approaches in this field.

## Author contributions

All authors contributed to the writing of the first draft manuscript. The aim and conceptualization of manuscript PF-E and VZ-C. PF-E, CI, AO-O, CH-R, IM-H, DO-S, VG, and VZ-C performed manuscript review and editing. All authors contributed to the article and approved the submitted version.
